# Towards story-based classification of movie scenes

**DOI:** 10.1371/journal.pone.0228579

**Published:** 2020-02-11

**Authors:** Chang Liu, Armin Shmilovici, Mark Last

**Affiliations:** Department of Software and Information Systems Engineering, Ben-Gurion University of the Negev, Beer-Sheva, Israel; University of Arizona, UNITED STATES

## Abstract

Humans are entertained and emotionally captivated by a good story. Artworks, such as operas, theatre plays, movies, TV series, cartoons, etc., contain implicit stories, which are conveyed visually (e.g., through scenes) and audially (e.g., via music and speech). Story theorists have explored the structure of various artworks and identified forms and paradigms that are common to most well-written stories. Further, typical story structures have been formalized in different ways and used by professional screenwriters as guidelines. Currently, computers cannot yet identify such a *latent narrative structure* of a movie story. Therefore, in this work, we raise the novel challenge of understanding and formulating the movie story structure and introduce the first ever story-based labeled dataset—the Flintstones Scene Dataset (FSD). The dataset consists of 1, 569 scenes taken from a manual annotation of 60 episodes of a famous cartoon series, The Flintstones, by 105 distinct annotators. The various labels assigned to each scene by different annotators are summarized by a probability vector over 10 possible story elements representing the function of each scene in the advancement of the story, such as the *Climax of Act One* or the *Midpoint*. These elements are learned from guidelines for professional script-writing. The annotated dataset is used to investigate the effectiveness of various story-related features and multi-label classification algorithms for the task of predicting the probability distribution of scene labels. We use cosine similarity and KL divergence to measure the quality of predicted distributions. The best approaches demonstrated 0.81 average similarity and 0.67 KL divergence between the predicted label vectors and the ground truth vectors based on the manual annotations. These results demonstrate the ability of machine learning approaches to detect the narrative structure in movies, which could lead to the development of story-related video analytics tools, such as automatic video summarization and recommendation systems.

## 1 Introduction

A story is a report of connected events—real or imaginary, and an event comprises a series of consecutive interactions between characters and objects (including other characters, nature, etc.) [1]. A story plot is defined as a sequence of events that have logical connections with each other [2]. Literature researchers have identified structural similarities between different stories: they claim that most stories can be attributed to a fairly *small* set of unique plots [3–5] (i.e., *narratives*—generalizations of frequently re-occurring sequences of events) about a *few* archetypal characters [6].

A screenplay is a manifestation of the art of visual storytelling [7]. While in a novel a conflict or an emotion may take place inside the readers imagination, inspired by the written text, a movie is an audio-visual medium, which can reveal the characters’ conflicts and emotions audio-visually (e.g., via a combat scene or an angry tone of voice).

While storytelling is a form of human artistic expression, we conceive of story writing and, in particular, scriptwriting for TV programs and movies, as built upon a few fairly known generic principles—a narrative structure [1, 7]. Creating quality narrative work that can captivate a large audience is a complex art. Aristotle’s *Poetics* is the earliest surviving work on dramatic and literary theories in the West. His *three-act form*—the broad notion that all dramas have a beginning, a middle, and an end, and that these parts obey some proportion law with regard to each other—is still prevalent in the modern movie industry. This structure, as applied to movies, has come to mean Act One, Act Two, and Act Three. Each act has its own characteristics: Act One introduces the character(s) and the premise—what the movie is about; Act Two focuses on confrontation and struggle; Act Three resolves the crisis introduced in the premise. Various plot devices, intended to intensify conflict, develop characters, and propel the plot forward, operate in each act.

Current video processing techniques focus on understanding low-level information such as objects or motion tracks, and can hardly provide any high-level insights directly from the video [8]. Machine-learning-based action recognition and localization aim to identify a given action and to localize its temporal period in the video. For short video clips (of about 10 sec.), the current state-of-the-art for the Kinetics-600 benchmark dataset of annotated videos is about 70% mean average precision for action recognition [9]. However, reliable automated methods for understanding high-level narrative elements of a movie storyline, such as falling in love with somebody or fighting for justice, have not, as yet, been developed.

The purpose of this research is to enhance state-of-the-art video analytics algorithms with story understanding capabilities. The novelty of the proposed approach lies in the use of knowledge gained from screenwriter’s guides (such as [10]) to impose a latent narrative structure on a given story (e.g., the setup in Act 1, the confrontation in Act 2, and the resolution in Act 3), which would leverage current video analytics algorithms. We envision this research as an important step towards developing a video understanding system that would receive a movie (or other narrative video) as input and generate useful textual summaries of the movie content at several abstraction levels, perceived by humans as readable, informative, coherent, and concise.

The proposed video understanding methodology is evaluated on *The Flintstones*, an American TV series of 166 animated cartoons. Each cartoon is about 25-minutes long, and presents a relatively simple, yet not too trivial, story. *Gupta et al*. have recently released a dataset of 25,184 densely annotated 3-seconds-length video clips extracted from the Flintstones cartoons with information such as object location, character identification, and textual description for each clip, for the purpose of learning to synthesize new clips [11]. In this paper, we present the Flintstones Scene Dataset (FSD). The dataset contains 60 episodes of The Flintstones, which are split into 1,569 scenes. The story-related function of each scene was manually labeled by at least three different human annotators as one of 9 possible key story elements defined in screenwriting guidelines (such as Climax 1, Obstacle). *This is the only dataset we know of that tags narrative videos by their story-related elements. Papalampidi et al. attempted to understand the narrative structure of movie synopses and screenplays (text only) by identifying turning points based on professional screenwriting knowledge* [12]; *this is a closely related research to the work presented in this paper*.

Under the premise that the detection of the latent structure of a story is essential for its comprehensibility, we constructed several story-related features and used state-of-the-art multi-label classification algorithms to predict the probability distributions of story-related class labels for each scene. Evaluation was performed by measuring the cosine similarity and the KL divergence between the predicted label probability distributions of each scene and the ground truth distributions (computed from human annotated class labels). To the best of our knowledge, *this is the first attempt to learn a latent story structure from videos via machine learning*.

The rest of this paper is organized as follows: Section 2 presents the story model we use in this work; Section 3 introduces the characteristics of the proposed scene classification dataset; Section 4 describes the methodology we used for data pre-processing, feature generation and scene classification; Section 5 presents the evaluation metrics we used, as well as the three proposed evaluation baselines; Section 6 discusses the results; Section 7 covers the related video analysis literature and, finally, Section 8 concludes with some discussion.

## 2 Story model

There are many studies that propose a template for creating quality narrative work for books [1, 2] and for screenplays [7, 10]. *Chris Huntley* compares between seven different story models for script writers in his blog [13]. In this paper we assume that a typical screenplay follows the “three-act structure” of [7]:

- **ACT 1 is about Set-up**. The scriptwriter sets up the story, establishes characters, launches the dramatic premise (what the story is about), illustrates the situation, and presents the relationships between the main characters.
- **ACT 2 is about Confrontation**. This act is all about conflicts between the main character’s dramatic need (what he/she wants to gain or achieve) and the obstacles he/she encounters.
- **ACT 3 is about Resolution**. This act presents the “solution” of the story: does the main character win or lose? Live or die? Get married or not? Return home safely or not? The results of the main character’s pursuit of his/her need/desire are presented in this act.

The scene is the basic unit in a screenplay. Typical scenes either contain a story element that advances the story towards its conclusion, or convey information about the characters and their situation. The relation between the three above-listed acts and scenes is hierarchical: if we regard the three acts as the spine of a screenplay story, then the key scenes form its “skeleton”. Considering that a screenplay typically has 40 to 60 scenes, there are multiple scenes that are not essential for understanding the core story. Those other scenes, which form the “flesh” of the movie, may introduce subplots, background information about the main characters, and scenes for enhancing the enjoyment of the audience (such as action scenes).

Various ideas have been proposed regarding how to craft the key scenes in the story [2, 10]. Though their definitions slightly vary, they mostly share the same basic view. Following their suggestions, we defined a list of key scene categories, listed below, which are expected to appear in most movies that conform to the “three-act structure”: *Inciting incident*, *Climax 1*, *Obstacle*, *Midpoint*, *Disaster*, *Crisis*, *Climax 2*, *Climax 3*, *Wrap-up*.

1. **Inciting incident**: The event that ignites the story and introduces the protagonist to the conflict that will need to be resolved in the rest of the story.
2. **Climax of Act 1 (noted as Climax 1 in the rest of the paper—as for Climaxes 2 and 3)**: The most important event of Act 1—it tells the audience what is the goal of the main character in this story, so that the audience will know, in general, what is the theme of the story, and what does the main character want to achieve. Climax 1 pushes the plot from Act 1 to Act 2.
3. **Obstacle**: Any difficulties the main character encounters while he/she pursues his/her need.
4. **Midpoint**: This is somewhere close to the middle of the story. In some types of story, this may be a “false victory”, where the main character thinks he/she has won or obtained what he/she wants. However, it is a “false victory” and the main character will figure out the truth later in the story. In some other types of story, the Midpoint may be a “false defeat”. Another feature of the Midpoint is **change**; it changes the situation of the characters, and diverts the story to the direction of the real end.
5. **Disaster**: This is not as general as an obstacle; a disaster is a critical event that may ruin the main character’s world (either physical or spiritual or both). It may crush faith or eliminate hope. The main character might stay in a low mood for a period of time until some events happen when he/she has to make the ultimate choice. A disaster usually changes the life conditions of the characters, but does not make a real difference regarding how the problem could be solved.
6. **Crisis**: A crisis is an event when the main character must make the ultimate choice. It will determine the rest of the story.
7. **Climax 2**: Here come the most intensive events within the story. In Climax 2, all puzzles are solved, all mysteries are uncovered, the main character finally gets rid of all the uncertainty and participates in the last fight. Climax 2 usually happens at the end of Act 2, and soon the plot will enter the last act.
8. **Climax 3**: Unlike Climax 2, where the main conflict is still in progress, in Climax 3, everything comes to an end. The audience will know whether the main character succeeds or not, and if the main character is going to finish his/her journey. Climax 3 is the most important event in Act 3.
9. **Wrap-up**: This element reveals the situation after the solution was obtained.

**Fig 1:**
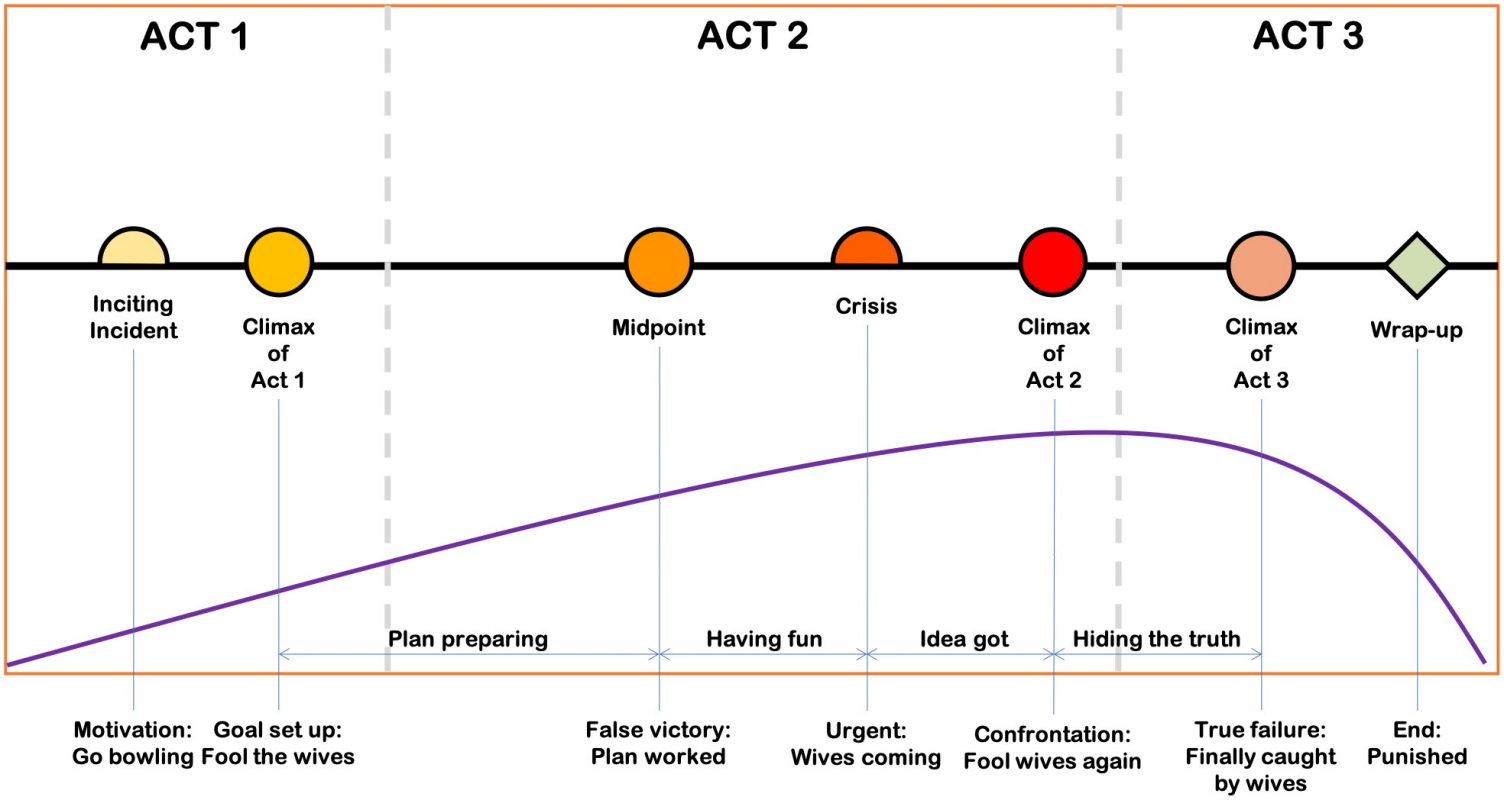
Example of applying the “three-act” model to the first episode of the Flintstones, season 1. Besides the notations of different story elements, the purple curve is used to represent the intensity of the story, which reaches its highest point at Climax 2, where the most dramatic event of the story takes place.

In the rest of this section, we present how to apply the story model to the Flintstones cartoons, and depict in Fig 1 the model application for an example story.

Most Flintstones episodes match the story model defined above quite well: a story in The Flintstones is usually about a conflict between Fred (sometimes with his friend Barney) and his wife/their wives (Wilma and Betty). The men trying to hide something embarrassing from their wives, who, eventually, will find out what the men are actually doing (e.g., the men want to go to the ball-game instead of taking their wives to the opera, by faking illness, see Fig 1). Typically, we have: (1) **Inciting incidents**, when the protagonist (usually Fred) is considering doing something embarrassing; (2) **Climax 1**, when the protagonist decides to execute his plan and hide it from his wife; (3) **Climax 2**, which is the confrontation between the men and their wives. To make the story more dramatic, two more elements are added: (4) **Midpoint**, a scene in which the protagonist faces his first major success (or defeat)—Fred thinks he will get away with his act without his wife noticing; and (5) **Climax 3**, when the wives eventually find out the truth concealed by their husbands. There could also be a few **Obstacles** that the protagonist has to face (such as the lack of money, unexpected traffic jam etc.), a **Disaster** that ruins the protagonist’s imagination of success, and a **Crisis** when he must come up with a solution, otherwise he will fail. Finally, after **Climax 3**, a **Wrap-up** ends the story by showing the aftermath of the protagonist.

An illustrative example of applying the previously described story model to one of the stories in the Flintstones cartoons is presented in Fig 1. It presents the story advancement of the first episode of The Flintstones, season 1. The summary of the story in this episode is as follows (available on Wikipedia at: https://en.wikipedia.org/wiki/List_of_The_Flintstones_episodes):

*On a Sunday, Fred fakes illness so he and Barney can get out of taking their wives to the opera, as the night coincides with a Bowling Championship. With the use of Barney’s homemade prehistoric helicopter as a means of escape, the two then join their bowling team for the tournament. They almost get away with their scheme, until loose-lipped Barney gives away their night’s activities by using a fake mustache he and Fred used earlier to try to trick their wives at the bowling alley*.

As illustrated in the graph, most of the key story elements exist in this short and relatively simple story. To some extent, a well written story should include those elements, no matter if its writer is explicitly aware of any story structure theories. However, since writing stories is highly dependent on the characteristics of individual writers, we expected to find stories which cannot be well described by the above architecture.

## 3 Dataset characteristics

Compared to cinema movie scripts, stories in animated cartoons are sometimes designed to be simple. By reading the episode summary available on Wikipedia (available at: https://en.wikipedia.org/wiki/List_of_The_Flintstones_episodes) and watching the Flintstones cartoons by ourselves, we found that their storylines are relatively simple. There is typically a single storyline in each episode, with a linear plot time (i.e., without flashbacks). More complex story models (such as “Save the Cat” [10]) include more situations (such as the B-Story class) that can accommodate for secondary plots and, therefore, handle more complex movie stories, such as the Simpsons cartoons. The plots in cartoons like The Flintstones are clear enough for humans to understand and more likely to fit in the story model we use; therefore, the important story elements should be relatively easy to identify.

We present the Flintstones Scene Dataset (FSD), extracted from 60 episodes of the cartoon series The Flintstones (the dataset and source code are published at: https://github.com/llafcode/The_FSD_dataset.git). The Flintstones is a famous American cartoon series, which takes place in a romanticized Stone Age setting, and follows the activities of the titular family, the Flintstones, and their next-door neighbors, the Rubbles (who are also their best friends). The stories in different episodes of the series are simple and usually independent (no cross-episode plot). The dataset consists of 1,569 scenes from the original cartoon episodes, meta-data of the scenes (e.g., scene duration, start and end timestamp etc.). We used a third-party software, PySceneDetect, for the scene splitting step. 105 undergraduate engineering students in the same data science course were invited to annotate the scene labels (the involvement of human participants in this annotation experiment was approved by the Human Subjects Research Committee in Ben-Gurion University). Each annotator was assigned 4 episodes for annotation: one test episode (episode 1 season 1) that all annotators were required to annotate (for the purpose of practicing the annotation process and receiving feedback) and three distinct episodes as the true annotation task. The annotators were free to manage their own working time, within one week after registering to undertake the task. To avoid tiredness, they were not required to annotate all the episodes in a single session. The annotation task was done with a single Google Form per episode, in which the annotator was confronted with a story-related question and one drop-down box per scene to choose its label. All the materials that the annotators needed were sent to them via a link to a Google Drive folder, in which they could find 1) the annotation guideline file, 2) four annotation forms, 3) the four full episodes they were assigned and 4) all the scenes for those four episodes.

We used Fleiss’ Kappa [14] to measure the across-episode consistency among human annotators and removed 57 suspected negligent episode annotations. Eventually, each episode was annotated by at least three different annotators (up to seven annotators per episode) and the mean Kappa score for the 60 episodes was 0.40, which can be interpreted as *Fair* according to [15]. Moreover, among the total 1,569 scenes, 625 scenes were labeled as Non-key and 92 as key labels by all annotators. Thus, there was full agreement on 717 scenes, which were 45.7% of all scenes. Besides, there were 735 scenes where more than half of the annotators made the same choice (374 on Non-key labels and 361 on key labels). In other words, we eventually obtained 1,452 scenes (92.5%) where the majority of human annotators reached agreement (999 Non-key scenes and 463 key scenes). This indicates that the human agreement was relatively high. Table 1 presents some descriptive summaries of the dataset.

**Table 1:** Dataset summary.

	Min	Max	Ava	Median
**Episode duration (mm:ss)**	25:04	26:25	25:47	25:06
**# of scenes per episode**	10	53	26.15	24
**Scene duration (mm:ss)**	00:02	07:48	00:55	00:30
**Kappa**	0.21	0.68	0.40	0.37

The Kappa score indicates that the task of labeling movie scenes with their roles in the story is subjective, because narrative works may be understood differently by different viewers. For example, a scene with humorous conversations might delight and amuse some viewers, but bore the others. People may agree that the story has a general shape, yet, for some specific events, it is hard to get consistence from everyone. Thus, instead of assigning a single label for each scene in the FSD dataset via choosing the majority labels, we decided to apply probabilistic labeling for each scene. Each label is presented as a distribution over all possible labels. Specifically, let {(*l*_*i*_, *m*_*i*_)|*i* = 1…*n*} be the labels and their corresponding annotation votes for one scene, where *i* is the ID of class labels. Then, the final label of this scene is:

$$\begin{array}{c} \left\{\left(l_{i},p_{i}\right)|p_{i}=\frac{m_{i}}{\sum m}\right\} \end{array}$$ (1)

where *l*_*i*_ is the *i*^*th*^ class label, and *p*_*i*_ is the probability of this label being annotated for the scene.

**Fig 2:**
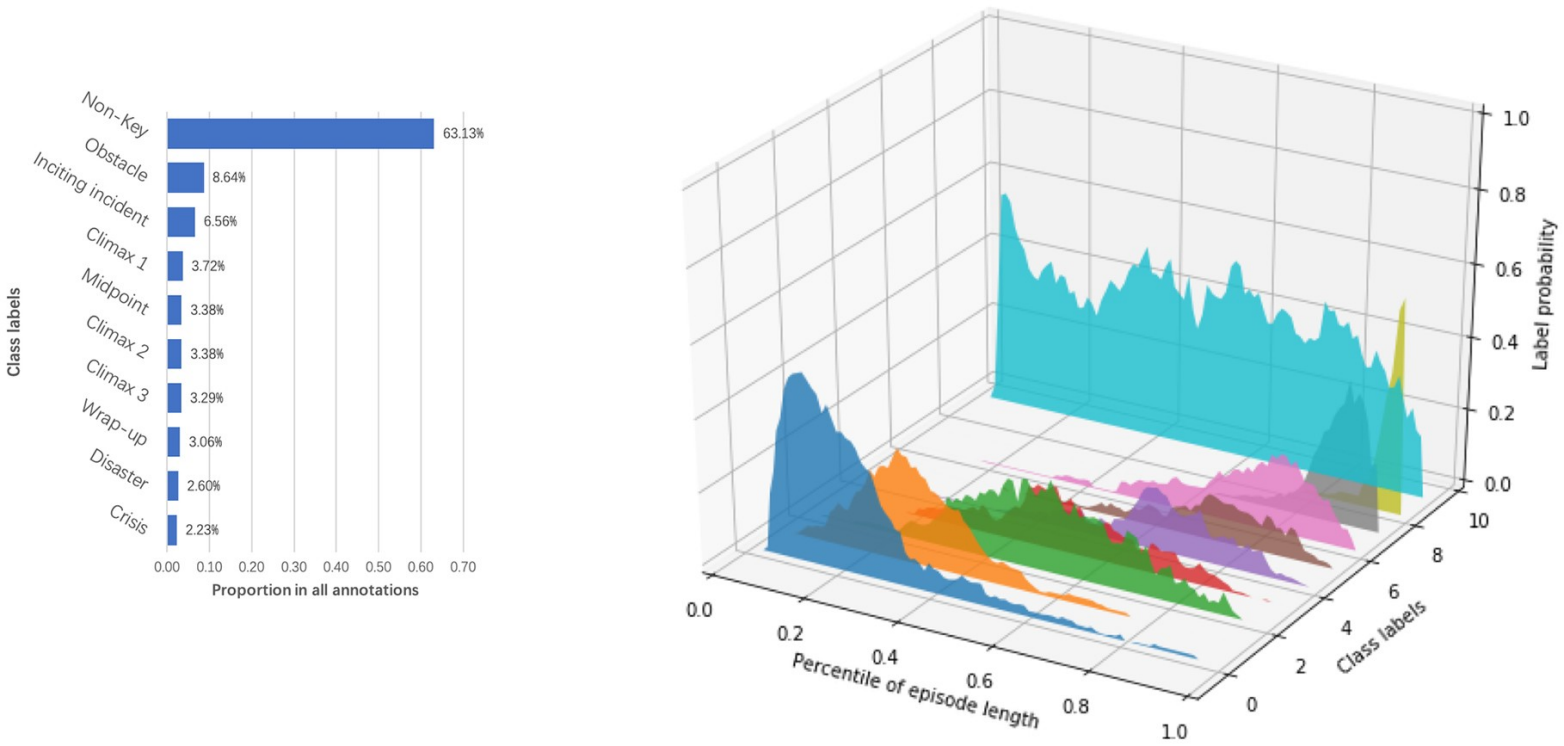
Label distribution of the FSD dataset. **Left**: label histogram after over-sampling. Within all annotations from 105 annotators, 63.13% of the annotations are *Non-key*. *Obstacle* and *Inciting incident* are more likely to be chosen among the key elements, while the others are almost uniformly distributed. **Right**: temporal label distribution over all episodes. On the class label axis, each tick represents a label: 0—*Inciting incident*, 1—*Climax 1*, 2—*Obstacle*, 3—*Midpoint*, 4—*Disaster*, 5—*Crisis*, 6—*Climax 2*, 7—*Climax 3*, 8—*Wrap-up*, and 9—*Non-key*.

Standard machine learning software is not adapted to probabilistic labels. To be able to use standard machine learning software, we “fooled” it by producing an over-sampled dataset that represents the original probabilistic labeling: we over-sampled the dataset so that we duplicate each scene to have 60 copies with one single label per copy, while keeping the ratio of different labels per the original distributions. A detailed explanation of the over-sampling process is presented in Appendix 3. Fig 2 (left) presents the final label histogram of the proposed dataset. It is computed from all the clean annotations of the 60 episodes from 105 annotators after over-sampling.

Our dataset is constructed on the assumption that the classic and widely used story structure, the “three-act” structure as described in previous section, was used to create all episodes in this TV series. We wrote the guidelines and instructions for the annotation experiment according to this theory, and, therefore, it can be expected that the obtained key scenes should be at relatively fixed locations. For example, Climax 1 should be around the first third of the story, while Climaxes 2 and 3 are expected to be closer to the end of the story, since their functions are to reveal and solve the conflict. More details of the process of data and annotation collection can be found in Appendix 1 and Appendix 2, as well as the guideline documents we provided to the annotators in S1 File. Fig 2 (right) demonstrates the proportion of each label in every percentile of the episode length. It is computed by averaging the probability of each label at each percentile of the episode length in the dataset among all annotated episodes. The original scenes in the dataset were aligned to percentiles using the start timestamp of each scene. The obtained distribution not only indicates that the annotators have followed the guidelines correctly, but also illustrates the temporal characteristic of the class labels. The location density for each label was caused by disagreement between annotators, the difference between the 60 stories, and the scene duration. The relatively temporally concentrated class distributions indicate that most of the stories inside the episodes in the FSD dataset conform to the story model. Moreover, Fig 2 (right) indicates that the actual distribution of labels assigned by humans to various scenes is temporally concentrated in the section of the episode roughly corresponding with Fig 1, which justifies using it as ground truth in our experiments.

## 4 Story-related scene classification

As the scenes are labeled with a distribution over all possible labels, we expect the output of our model to be a label probability vector as well. In this section, we introduce the methods we used to predict such a vector for a given scene. First, we propose three baseline approaches for the purpose of comparison that do not involve learning. Then, we introduce a machine learning approach using temporal and story-related features.

### 4.1 Baseline approaches

#### 4.1.1 Most common label vector

As a simple baseline, we assign the label *Non-key* to all scenes, as *Non-key* is the majority class. Specifically, we set the output vector with all elements as 0 except for the probability of *Non-key*, which is set to be 1.0.

#### 4.1.2 Label distribution vector

A more suitable random labeling strategy is to choose labels based on the proportion of each label in the entire dataset. Therefore, we use the label distribution after over-sampling (as shown in Fig 2 left) as the output vector, in which each element is the proportion of each label, representing the probability of that label being chosen for a given scene.

#### 4.1.3 Temporal label distribution vector

The previous two approaches did not take into account the temporal nature of the labels. With the temporal baseline, we predict the label probability vector for each scene based on the relative location of that scene. In particular, for a given scene with a start timestamp *p*_*s*_ and an end timestamp *p*_*e*_ (both timestamps are converted to a percentile by dividing by the episode duration), we take the area between *p*_*s*_ and *p*_*e*_ from the distribution shown in Fig 2 (right), and compute the average probability of each label within that area. The output probability values do not need any normalization, as they naturally sum up to one because for each scene the original probability distribution among all labels sums to one, leading to the same phenomenon when we compute the temporal label distribution for each percentile of the movie time. In other words, given the temporal label distribution, for any scene interval the estimated probability vector sums to one.

#### 4.1.4 Logistic regression

Multinomial logistic regression is a widely used model for multi-class classification tasks, and we use it as an additional baseline. As required by the typical classification algorithms that include logistic regression, the data labels should be discrete class labels; therefore, the oversampled data (as described in Section. 3, in which each sample has a single label) is used during training. The testing phase is done on the original data using similarity as the evaluation metric. For this baseline approach, we train the classifiers with all features.

### 4.2 Feature engineering

The story model we use in our paper (see Section. 2) imposes a latent narrative structure on movies. One may presume that features that follow the elements of that structure will improve the scene classification performance. In the following sub-sections we describe in detail the features we used for classification. The full list of features is shown in Table 2.

**Table 2:** Full feature list used for story-based scene classification.

Feature Set	Name	Type	Description
**Basic**	*i*	int	scene ID
	*dur*	float	scene duration (seconds)
	*t*	float	start time (seconds)
	*close*_*beg*_*id*	float	closeness to the beginning, 1/*i*
	*r*_*id*_*loc*	float	*i*/*n*, *n* is number of scenes in the episode
	*r*_*t*_*loc*	float	*t*/*len*, *len* is the duration of the episode
	*sec*	int	section id, *sec* ∈ [1, 2, 3, 4, 5]
**Visual two clocks**	*visual*_*clock*_*drift*	float	the clock drift at the current scene
**Character network**	*protagonist*_*appear ava*_*scores*	bool float	1: the episode’s protagonist appears in the current scene the average character scores

#### 4.2.1 Basic features

The basic features represent the sequential order of scenes in a movie episode. Specifically, inspired by *Edmundson (1969)* and *Baxendale (1958)* [16, 17], the scene’s ordinal location *i* and its closeness to the beginning of the episode (1/*i*) are the used features here. In addition, scene duration (noted as *dur*, in seconds) and start time (noted as *t*, in seconds, which means the current scene starts at the *t*-th second of its episode), are also used directly as basic features. Moreover, the relative location of each scene in the episode is computed in two distinct ways (with both the scene ID and the scene start time), yielding two features: *r*_*id*_*loc* = *i*/*n* (where *n* is the total number of scenes in each episode) and *r*_*t*_*loc* = *t*/*len* (where *len* is the total length of each episode). Another feature used to represent the location of a scene is the “quantile section” to which it belongs. Guidelines for professional scriptwriters recommend to divide the story into specifically sized parts, and some events should happen in specific parts. For example, a story can be partitioned into 5 parts. An Inciting incident usually happens in the first part, while a Midpoint should happen in the third. In this work, we divide an episode into 5 equal-length sections and use the section ID (*sec*) as the last basic feature.

#### 4.2.2 Story-related features: The two-clocks

An important observation regarding time perception, made by psychologists and neuroscientists, is that time flows differently in different emotional situations. This is because our biological clock usually adapts to the environment, causing the shrinking and expansion of the perceived time. In the context of narrative work, the perception is that time moves faster when many activities are involved. This idea has been used by *Lotker* to detect time perceptions in social networks and critical events in Shakespeare play scripts [18]. In his work, *Lotker* raised the question of how to measure the time perception within an evolving structure and proposed the *two-clocks* theory as a solution. In the theory, two clocks are modeled: (1) the event clock measures the regular time-flow (number of utterances by the characters) and (2) the weighted clock measures the time of an event (number of words every time a character speaks). By finding the *clock drift* (maximum difference) between the two clocks, the critical event within the play is detected. In our previous work, we adapted the *two-clocks* theory to the domain of video analytics, and tested it on the first season of the Flintstones cartoon series to identify turning points within the stories [19] using only the subtitles. In this work, however, we utilize the visual information, rather than text, to implement the *two-clocks* theory and craft story-related features.

An important scene in a movie usually contains more visual events, leading to a higher visual complexity of that scene. It is possible to model the two clocks as (1) an event clock that represents the regular time flow and (2) a weighted clock that represents a measure of the visual complexity of a given scene. The visual complexity of a scene can be measured by the storage space its video file occupied. We stored all video files in .MP4 format which compresses video files according to the complexity of the frames. A more complex sequence of frames (e.g., one which contains fast visual events such as object motion) cannot be compressed very well and will need more space per video frame than a static image. Practically, the two clocks tick simultaneously but increment in different ways: the event clock ticks by one for each scene and the weighted clock ticks by the number of bytes the video file of that scene occupied in the system. After the two clocks are constructed, the difference between them at each scene is computed by the following gap function:

$$\begin{array}{c} \delta =NC_{w}-NC_{e} \end{array}$$ (2)

where *NC*_*w*_ and *NC*_*e*_ are the values of the normalized weighted clock (*C*_*w*_) and the event clock (*C*_*e*_) (min-max normalization is used) in a given scene, and *δ* is the difference between the two clocks in that scene. Then, the clock differences are used as the *visual_clock_drift* feature, as shown in Table 2.

The importance of action scenes with a lot of motion, compared to conversation scenes, is not universally agreed, because it depends on the movie genre and the story itself. Yet, unlike the theatre, movies are expected to “show rather than tell”. Nevertheless, the two-clock feature is useful because it not only represents the compression rate per scene, but, more importantly, reflects how the compression rate changes over the movie time, by computing the difference between the two clocks. Therefore, if there is a sudden change in the trend of the second clock, either from fast to slow or vice versa, we expect an important event to be happening.

#### 4.2.3 Story-related features: The character network

A simple story is usually driven by the events that happen to the main character, also known as the protagonist. It is reasonable to assume that the importance of a scene in a movie is related to the likelihood of the appearance of the protagonist and other important characters. Basically, we expect that the protagonist will be likely to appear in each key scene within the story. *Tran et al*. proposed the co-occurrence character network (CoCharNet) to represent the relationships between characters, as well as an approach to detect the protagonist within a movie by measuring the centrality and weighted degree of each node within the network graph [20]. In our work, we follow their definitions to build one character network for each episode and to extract story-related features from the network. Each node in the network represents a character and the thickness of each edge connecting two nodes represents how often these two characters co-occur in the same scene. An importance score is computed and assigned to each character to reflect their importance within the network. Specifically, the importance score of the *i*^*th*^ character $S_{c_{i}}$ is defined as:

$$\begin{array}{c} S_{c_{i}}=\frac{1}{3}\left(E_{c_{i}}+C_{c_{i}}+W_{c_{i}}\right) \end{array}$$ (3)

where *c*_*i*_ is the *i*^*th*^ character in the network, $E_{c_{i}}$ is the eigenvector centrality of *c*_*i*_, $C_{c_{i}}$ is the closeness centrality of *c*_*i*_ and $W_{c_{i}}$ is the weighted degree of *c*_*i*_.

To construct a character network it is necessary to correctly identify the character appearance events in the videos. Ideally, computer vision based approaches could be used for this task; however, such tools (e.g., Microsoft Video-Indexer) for detecting character faces are usually trained on real-life videos rather than annotated cartoons, and hence they are not effective in cartoon movies [8]. Therefore, for the construction of the character networks, we made use of the dataset of *Gupta et al*., which consists of 25,184 densely annotated 3-second video clips taken from the Flintstones cartoons. The annotations in that dataset include the names of the characters that appeared in each clip; hence, we can use the video clips in that dataset for dense (at least one clip per scene) character appearance sampling over the entire episode, and construct an episode-wise character network based on the character annotations within that dataset.

**Fig 3:**
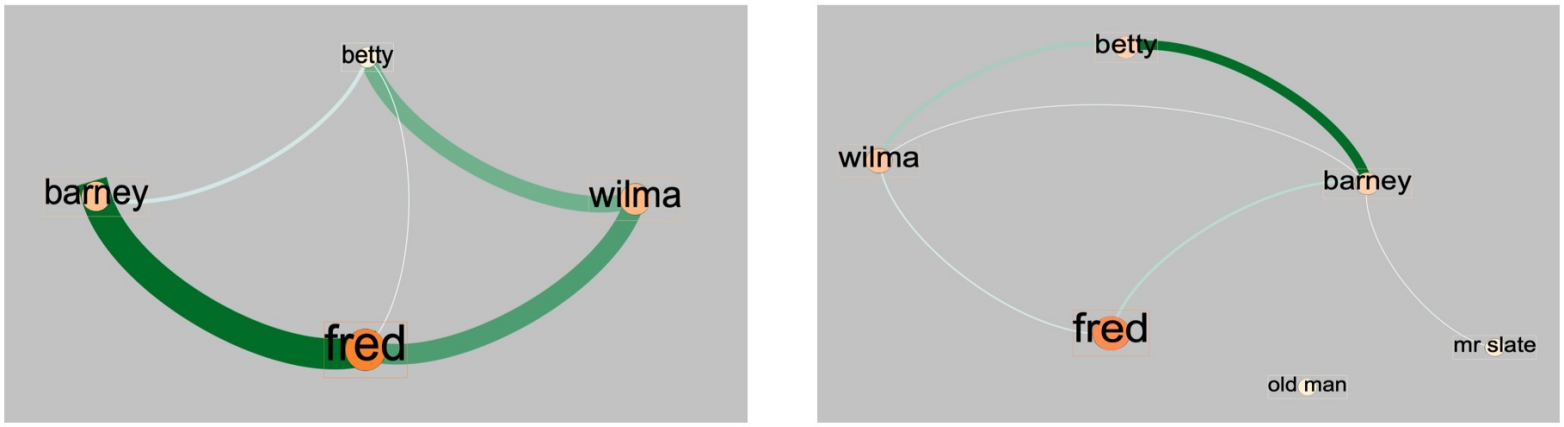
The examples of co-occurrence character network built for feature extraction. **Left**: the character network for episode 13 season 1. It is a story about Fred and Barney secretly planning to buy and run a drive-in restaurant. **Right**: the character network for episode 22 season 1. It is a story about a company manager who closely resembles Fred and was mistakenly regarded to be Fred by the other three regular characters. Meanwhile, Fred, mistaken as that company manager, was taken to the company by the company employees.

Two examples of the character network are presented in Fig 3. They are relatively simple because the number of characters in the stories is small. In episode 13 (Fig 3, left), since Fred and Barney have a secret plan, they appear together in most of the scenes, which leads to the thickest edge being between Fred and Barney. There were several times when Fred was almost caught by Wilma in the story, so this relation is also represented in the network where the edge between Fred and Wilma is the second thickest. On the other hand, in episode 22 (Fig 3, right), Fred was mistakenly considered to be the manager of a company whose manager visually resembles Fred. This is the reason why Fred showed up alone in most scenes in that episode. From the node size of Fred in the episode’s character network, it can be figured out that Fred is the main character, but the thin edges between Fred and the others reflect their weak relationships in this story.

As presented in Table 2, two features are extracted from the character network. The first is *protagonist*_*appear*, and is a binary feature which represents the protagonist’s appearance in a given scene. The protagonist is the character with the highest importance score ($S_{c_{i}}$) (most screen time) in the entire episode. In addition, we also use the average importance score of all characters in each scene as the second feature (*ava*_*s*_ *cores*).

### 4.3 Classification

We use one of the classical multi-label learning strategies—*one-vs-all* classification—during the training process. Conventional one-vs-all classification (also known as one-against-all, one-vs-the-rest) turns the problem of multi-label classification into an aggregation of binary classification tasks by learning one classifier per class, and all classifiers vote for the final prediction. In our work, however, we expect a 10 dimensional label probability vector, in which each element is the probability of the corresponding label for that scene, as output for each input scene.

## 5 Experiment

### 5.1 Evaluation metrics

Considering that the ground truth labels and the output of the model are both vectors of probabilities, we measure the performance of the model by calculating the cosine similarity between the output vector and the ground truth vector. For a given scene, the cosine similarity is computed as:

$$\begin{array}{c} S\left(y\_pred,y\_true\right)=\frac{y\_pred\cdot y\_true}{\left||y\_pred||*||y\_true|\right|} \end{array}$$ (4)

where *y*_*pred* is the predicted probability vector and *y*_*true* is the ground truth vector.

In addition, Kullback-Leibler divergence (abbreviated as KL divergence) is also used as an evaluation metric, since it measures the amount of information lost in the estimated distribution compared to the true distribution. Specifically, the KL divergence of scene *i* is computed as:

$$\begin{array}{c} KL_{i}=\sum p_{k}\ln\frac{p_{k}}{q_{k}} \end{array}$$ (5)

where *p*_*k*_ and *q*_*k*_ are the probability of the *k-th* label in the true and estimated distributions, respectively.

### 5.2 Experiment design

We evaluated the proposed baseline approaches, as well as the machine learning model, on the FSD dataset, which consists of 1,569 scenes from the Flintstones cartoon series, labeled with probability distribution vectors of all possible labels. In order to train a classification algorithm, we over-sampled the dataset to a single label per scene by duplicating all instances in the dataset to 60, which is the least common multiple of the different annotations within the dataset, while keeping the ratio among all labels constant. We obtained 94,140 instances after over-sampling with a single label per instance.

In order to retain the episode groups of scenes and avoid overfitting, we applied leave-one-group-out cross validation (cv) to evaluate the overall performance. The FSD dataset consists of 60 episodes; therefore, we created 60 groups by assigning one episode per group, regardless of how many scenes there are in the episode. In each cv iteration, a new model is trained on 59 groups and tested on the remaining group. Only training data (59 episodes) will be over-sampled; testing data (1 episode) will be used directly with the original probabilistic labels. The final score of the model performance is the average cosine similarity and KL divergence of all the scenes within the test episode, and the reported results in this paper will be the average similarity/KL divergence of the 60 cv folds. The leave-one-group-out cross validation method was applied to all baseline approaches (except for the most common label vector baseline) and the proposed XGBoost model.

We evaluated the performance of all approaches in two ways: (1) scene-wise performance—for each scene in one episode, the similarity/KL divergence between the true and estimated probability vectors was computed, and the average scene similarity/KL divergence results of all the scenes in this episode was taken as the episode’s similarity/KL divergence; (2) label-wise performance—in order to measure the performance for each class, respectively, for each episode, we computed the two scores (cosine similarity and KL divergence) for the true and estimated probabilities of each label in all scenes, and for each metric we obtained ten values representing the performance of ten labels.

We used XGBoost [21] as the classifier for each class. The XGBoost classifier won most of the recent data-mining competitions before the introduction of Deep Neural Networks. The implementations of the algorithms used in this work are based on the distributed Python implementation of XGBoost [22], and scikit-learn, a widely used machine learning library for Python [23]. We used the default parameter settings provided by the implementations (Table 3).

**Table 3:** XGBoost parameters.

Parameter name	Value
**objective**	binary:logistic
**n_estimators**	100
**gamma**	0
**learning_rate**	0.1
**max_depth**	3
**max_delta_step**	0
**min_child_weight**	1

## 6 Results and discussion

We compared the performance of different approaches and present the results in Table 4. The cosine similarity and KL divergence between the predicted vector and the ground truth vector are used for evaluating the performance of different approaches. Similarity close to 1 and KL divergence close to 0 mean that the difference between the vectors is small, indicating that the prediction is more precise. The scores presented in the table are the average similarity/KL divergence over 60 folds cross validation, and the confidence interval is the standard error of the mean of the 60 scores multiplied by 1.95. A paired t-test was run to measure the significance of the improvements; although the specific p-values are not presented, we denoted any statistically significant improvements with asterisks (*).

**Table 4:** Scene-wise performance of different approaches. We demonstrate the average similarity and KL divergence, with confidence interval (C.I.), over 60 folds cross validation. The confidence interval is computed by average similarity/KL divergence ±1.95* standard error of the mean. (*) denotes the statistical significance (by applying paired t-test).

Method	Similarity C.I.	KL divergence C.I.
Most common vector	0.69±0.03(*)	8.81±0.66(*)
Label distribution	0.71±0.03(*)	1.09±0.06(*)
Logistic regression (all features)	0.72±0.03(*)	1.05±0.06(*)
Temporal label distribution	0.73±0.01(*)	0.87±0.04(*)
XGBoost (basic)	**0.81 ± 0.02**	**0.66 ± 0.04**
XGBoost (basic + visualTwoClocks)	**0.81 ± 0.02**	**0.67 ± 0.04**
XGBoost (all features)	**0.81 ± 0.02**	**0.67 ± 0.04**

With only the knowledge of temporal label distributions, the temporal label distributions baseline outperformed the other baselines for both metrics. The XGBoost approaches further improved the average similarity by 0.08, and reduced the KL divergence by about 0.20; these are significant results, indicated by the p-values (paired t-test, respectively with the temporal label distribution baseline). The clear trend within the two metrics, i.e., KL divergence drops while cosine similarity increases, indicates the consistency between the two metrics. *Zhao et al*. evaluated a predicted probability distribution of human emotions evoked by images using KL divergence. They reported KL divergence from 0.4 to 1.2 (the lower the better), while ours ranged from 0.67 to 1.09. Hence, we believe that as a preliminary attempt to identify key story elements in cartoon movies, the obtained results are encouraging and promising.

We measure the importance of different features by running the built-in feature selection methods in the XGBoost implementation we used, which returns the weight of each feature, i.e., the number of times a feature appears in a tree. Three features out of ten were detected as the most important features. These are *r*_*id*_*loc*, *dur* and *r*_*t*_*loc*, in contrast to the three features which contributed the least (*sec*, *protagonist*_*appear* and *close*_*beg*_*id*). The main important features represent the location information of each scene, and the best story-related feature is *clock*_*drift* in *5-th* place in the feature ranks. It can be concluded that, although simple, the basic feature set well represents the temporal characteristics of the scenes, and has a strong influence (except for the *sec* feature) on the predictions. This finding also validates the story model so that the model can be used for extracting narrative structures from other movies.

Not being able to improve the scores does not necessarily mean that the story-related features are useless; instead, this might be attributed to the relatively simple story we attempted to understand. For example, a complex story, such as the story in a full length cinema movie, typically has more characters, sub-plots, and story-elements than in the Flintstones cartoons, and the protagonist usually does not appear in all the scenes. Thus, the appearance of the protagonist in a scene is a hint of the importance of that scene within the entire story. In the cartoons in our dataset, usually the same four main characters appear in most scenes, and no matter who is the protagonist, he/she may appear in most scenes, which leads to the feature *protagonist*_*appear* not being very informative. The low variance of character appearance over an episode also reduced the effect of the character scores feature, as most scenes have almost the same set of appeared characters.

**Fig 4:**
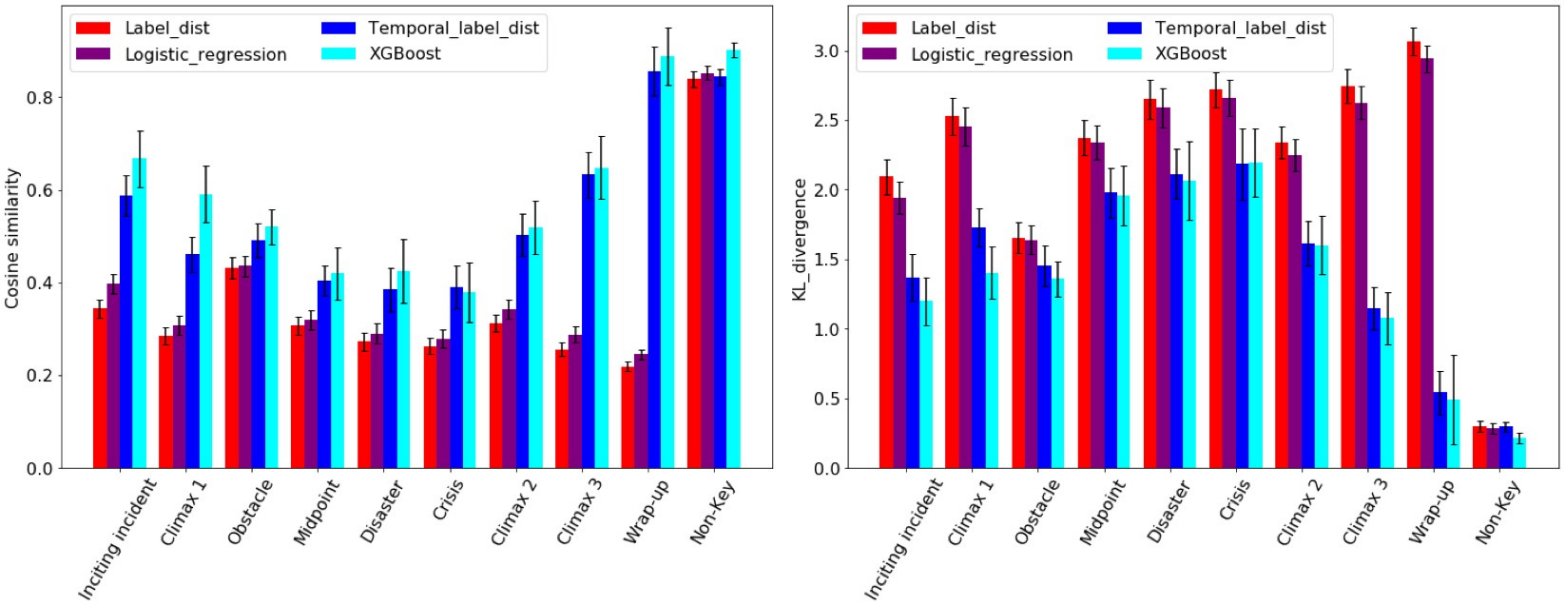
Label-wise performance. This figure demonstrates the label-wise performance of all approaches with two metrics: **Left**—cosine similarity and **Right**—KL divergence. For the XGBoost approach, we demonstrated only the results from the classifier trained with all features. The most common vector baseline is not included.

We further evaluated the performance of all the approaches for each label, and the results are displayed in Fig 4 (excluding the most common vector baseline). In this experiment, we trained the logistic regression classifier and XGBoost with all the features. In general, the temporal label distribution baseline and XGBoost outperformed the other approaches, and XGBoost was more accurate for detecting *Inciting incident*, *Climax 1*, *Obstacle* and *Non-key* (refer to S1 Table for more detailed scores and comparison). Similar to scene-wise performance, the two metrics showed their consistency on different labels, indicating that the start and end part of The Flintstone cartoons are easier to understand, while the key events that happen in the middle of an episode might be indistinguishable. A better reason might be the incompatibility between the story model we used and the relatively simple Flintstone cartoon stories, as the story theories we build upon were actually developed for 90+ minute cinema movies, rather than a 24 minute cartoon with a single simple story.

The scene splitting quality affects the final performance. The software we used for scene splitting detects scenes based on the visual difference between consecutive scenes, instead of any information related to the plot, sometimes resulting in scenes not being well split from the scriptwriting point of view. For instance, some scenes that contain *shots* (fast camera changes) are sometimes split into several scenes, while some distinct scenes are sometimes merged into one single scene. This inaccurate scene splitting reduces the quality of the annotation (it is likely that some annotators were confused by erroneous scene splitting and failed to select the best label for the scene) and increases the annotators labeling disagreement, and, hence, reduces the model accuracy. To reduce the effect of scene splitting errors, we used a label probability distribution of human assigned labels as ground truth labeling of each scene. Our experiments showed that closely located scenes have similar distributions (see Fig 2).

From our experience, the accuracy of state-of-the-art scene splitting tools, such as Microsoft’s Video-Indexer, is far from being perfect. Hence, scene classification methods should be as robust as possible to scene splitting errors. Evaluating the effect of the scene splitting quality on classification performance is part of our future research plans. Better overall performance can be expected if the scene splitting would be more accurate, or even done by professional human video-editing experts.

The proposed model performed worst on the 4^*th*^ episode of season 1 (S1E4), and the 26^*th*^ episode of season 1 (S1E26), obtained the episode similarity of 0.54 and 0.65, respectively. The Kappa scores for these two episodes are relatively high: 0.53 and 0.57, respectively, indicating that the annotators of those two episodes reached good agreement. By analyzing those two episodes carefully, we discovered that episode S1E4 is negatively affected by inaccurate scene splitting: only 16 scenes are detected and the variance of scene lengths is large (the shortest duration scene is 25 seconds while the longest duration scene is 216 seconds). Considering that the scene duration is an important feature for the classifier, such a high variance in scene duration prevented the algorithm from making appropriate predictions. On the other hand, Episode S1E26 was split into scenes properly and the annotators were in agreement, but the annotations reflected the fact that its story is not as typical as in other Flintstone cartoons: the classes *Disaster* and *Crisis* were chosen by the majority of annotators in this episode, while those two classes are rare among all episodes. It seems that the classifier is not capable of accurately detecting those rare classes.

Despite the existing limitations of the data generation and data annotation processes, the average similarity of 0.81 between the predicted label vectors and the ground truth vectors indicates that, as a preliminary solution to the story-based scene classification task, the current results are promising. Both the temporal label distribution and the machine learning approach with basic features demonstrate the strong temporal character of the labels. This implies that most of the stories within the proposed dataset conform to the story model quite well.

## 7 Related work

Automated video understanding research attempts to understand video content from different aspects: video captioning [24, 25] describes the main activity in a given video in a few sentences, as well as its agent (who), location (where), and temporal information (when) etc.; video summarization provides condensed and succinct representations of the input video in the form of skimmed videos [26, 27]; semantic video segmentation [28] detects, identifies and tracks objects, foreground and background within the video scenarios; video classification [29] categorizes large-scale video clips into diverse activity classes. The types of video studied by the community varies from self-made daily life amateur videos (e.g., from YouTube) through professional sports game recordings, to surveillance videos. Movies, as typical narrative videos, have been widely studied in order to address different challenges, such as movie summarization [20, 30], movie description generation [31, 32], and movie scene detection [33]. Current video understanding techniques excel at the clip level (a few seconds long videos) and, unlike our work, are not yet directed to narrated videos such as movies and TV series.

Question-answering is a popular task in the domain of Natural Language Processing (NLP) and has been adapted to images as well [34], but the domain of videos, even movies, is still new for this task. As a novel attempt for addressing the question-answering task on movies, *Tapaswi et al*. proposed a MovieQA dataset and benchmark results [35]. The questions collected in their dataset are story-related, and start from simpler “Who” did “What” to “Whom”, “Why” and “How”. Later, Na et al. improved the results on this task using neural networks [36]. A dataset called PororoQA (a Korean cartoon series) has been published recently [37] for story-related question-answering in cartoon videos. Movie stories question-answering provides impressive point-to-point understanding of specific elements and events in the movie plots, yet, it is hard to reveal how these events influence the stories.

Tran et al. proposed the idea of building a co-occurrence character network [20], which has been extended to an *affective character network*, representing the emotional relationship among characters. Based on this, *Lee and Jung* proposed a graph-style story model which reflects the story development [8]. Most recently, Lee and Jung proposed a character network embedding based method to detect subplots within a story. As a preliminary solution, they used K-nearest Neighborhoods for scene clustering based on scene correlations; these are calculated from the character network embeddings. Although these methods offer impressive results, their limitations are still obvious. The multi-modal features (such as frame color, brightness, audio and detected face boundary etc.) sometimes fail, because those features rely heavily on the movie genre or the production style [8], and, therefore, sometimes cannot represent the movie content appropriately. Unlike this research, their story structure (reflected by temporal graphs) is implicit: they know that a certain scene is important because of certain features it possesses, yet cannot explicitly explain its purpose in advancing the story (i.e., an “obstacle” scene).

Recently, *Papalampidi et al*. attempted to solve a similar task—automatic analysis of narrative structure in screenplays [12]. They adopted the screenwriting theory by *Hague* and defined five story elements that have to appear in a specific order [38]. Our work differs from theirs in two major aspects:

1. We build upon different and more elaborate screenwriting theories [2, 10], using nine story elements that may appear in a semi-flexible order;
2. We analyze narrative structure in movies rather than screenplays, which contain text only.

In our previous work, we extended *Lotker’s* two-clock theory [18] for detecting story turning points in The Flintstones’ subtitle text and achieved promising results on both turning points and key story element detection [19]. However, the ability of distinguishing key story elements is still lacking.

## 8 Conclusion

In this paper, we proposed a novel task: story-related movie scene classification, as an important step towards understanding the story structure within narrative videos such as movies, TV series or animated cartoons. We constructed the first benchmark labeled scene dataset, the Flintstones Scene Dataset (FSD), and presented promising results of using machine learning algorithms for the scene classification task. The evaluated algorithms were able to discover the sequential character of the key elements in the story model we used, which has been further verified by the temporal label distribution baseline and results with the basic feature set. The two-clocks features and the character network features did not contribute significantly to the classification performance.

We explored the possibility that different episodes in our dataset belong to different story structures, thus reducing the performance of the classification algorithms across the episodes. Considering the temporal label distribution presented in Fig 2 (right), we observe that there exist some story elements with a wide temporal distribution (such as “*2—Obstacle*” and “*3—Midpoint*”). Possible reasons for such wide distributions might be (1) multiple occurrences per episode (e.g., a story may have more than one *obstacle*); (2) difficult concepts (e.g., the annotators had a hard time understanding the concept of *Midpoint*), and (3) an episode not conforming to the story model (some episodes with very low Kappa scores indicate that the annotators are confused, meaning that the episode itself may not fit our story model well). It might be found that the classification performance would improve if the confusing classes were removed from the label set and only the most critical elements were kept (such as Inciting incident, and Climaxes 1, 2, and 3).

As a first attempt to learn the story structure of a narrative video, we believe that this work has opened a promising direction for video story understanding. This research can be extended to the more challenging problem of automatically understanding full length movies, by extracting a sequence of key story elements from the label distributions predicted for each scene. Future research can naturally follow our results by (1) adding high level story-related features such as automatically detected characters, objects, actions, and places etc.; (2) generating a story-aware summary (in a video or text format) of a given narrative video; (3) utilizing story-related scene classification to boost the performance of other story-related tasks, such as movie question-answering; (4) exploring alternative, more elaborate movie story structures; and (5) generating additional benchmark collections of story-based video annotations.

## Appendix 1

### Video data collection

We prepossessed the original videos of the cartoons before the annotation stage. For the Flintstones cartoon series, each episode starts with a short trailer of the episode (usually less than 1 minute), followed by the opening theme song (1.5 minutes), and at the end of each episode, there is an ending theme song (1 minute). We discarded those parts for each episode because they are not related to the story. The middle section of each episode (approximately 25 minutes long), which was the body of the cartoon, is split into scenes using a third-party software, PySceneDetect, which is publicly available at https://pyscenedetect.readthedocs.io/en/latest/. Though PySceneDetect tends to detect shots with its basic settings, there are parameters we can adjust to improve the splitting outcome. We combined its HSV (hue, saturation, value) based content-detector and RGB (Red, Green, Blue) based threshold-detector to obtain the best scene splitting results. The thresholds were empirically adjusted, respectively. We also limited the minimum length of a video clip to around 10 seconds so that we can avoid over-splitting caused by shot changes instead of scene changes. The detailed parameter setting of PySceneDetect is presented in Table 5. It is common to have fade-out and fade-in short clips (typically a less than 1.5 seconds—black clip) which leads the software to produce a video fragment during the scene splitting process. We manually removed all video fragments from the outcome scenes. Eventually we obtained 1,570 scenes from the 60 episodes.

**Table 5:** Parameters used in PySceneDetect.

Parameter	Value
Content-detector threshold	60
Threshold-detector threshold	12
Minimum output length	10 * fps

### Appendix 2

#### Annotation collection

**Fig 5:**
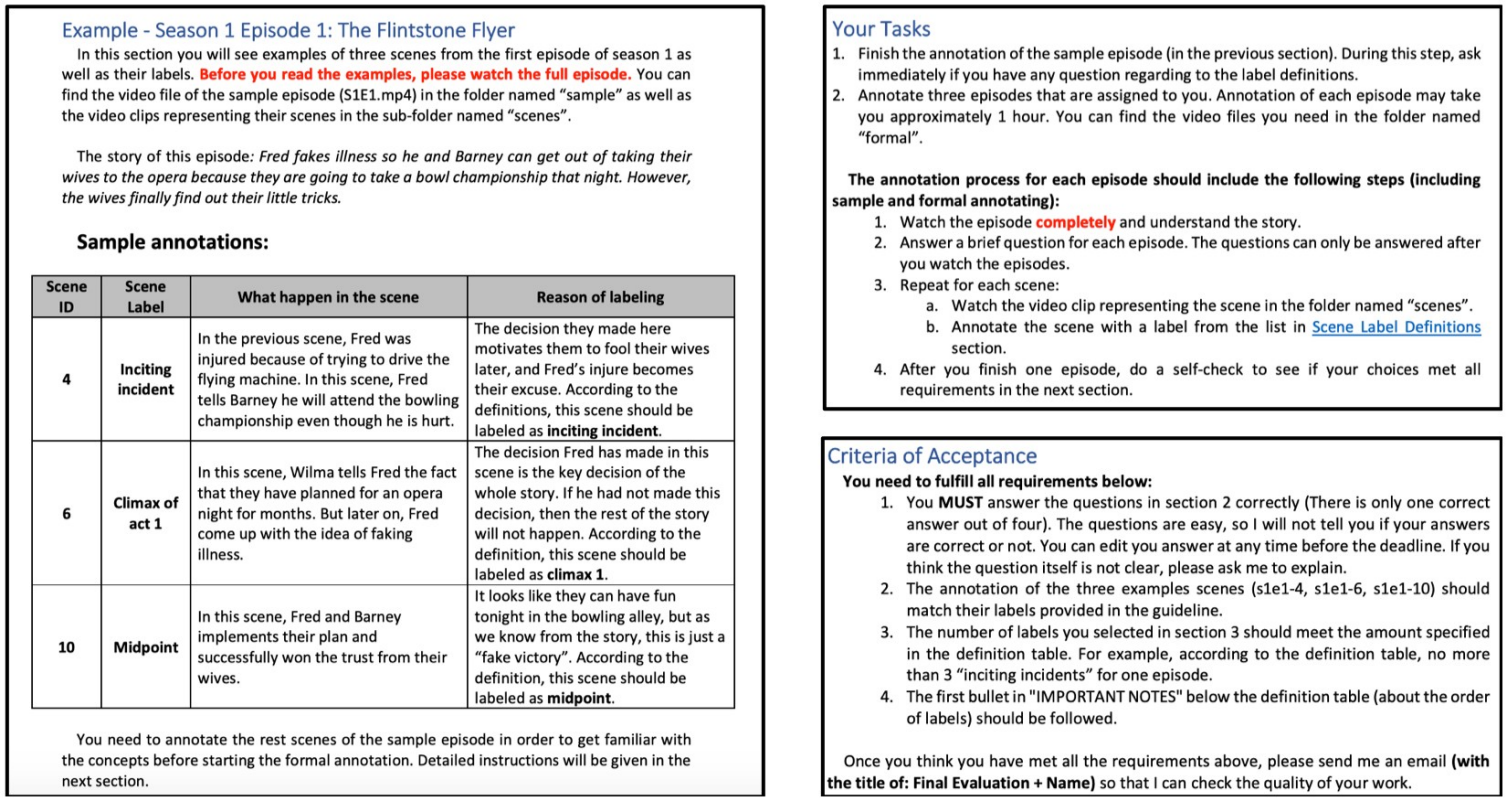
The annotation guidelines (snapshot). **Left**: story summary of the sample episode and annotation examples for three key scenes. **Top right**: the working procedure for each annotator. **Bottom right**: the requirements that annotators need to fulfill in order to be accepted.

In order to acquire reliable labels for the FSD dataset, we invited 105 human annotators for the labeling task. The invited annotators were 3rd year undergraduate engineering students in a data science course. Although they are not professional in the field of storytelling or narrative understanding, they were well trained and supported by our provided guidelines during the experiment. Detailed label definitions were provided in the guidelines to the annotators (the full annotation guideline document is submitted as support information in S1 File, Fig 5 demonstrates three snapshots of the guideline), and Table 6 presents a condensed version of the label definitions. In the guidelines, the annotators are introduced to the “three-act” story structure, and required to fulfill several requirements of the relative order of labels such as, *“Midpoint can only happen between Climax 1 and 2”, “Disaster can only happen after Midpoint”, and “Obstacle can happen anywhere after Climax 1*” etc. It is also necessary to meet the requirements about the amount limitation for each label, see the “Required Amounts” column in Table 6. We set up such requirements because the “three-act” structure is the theoretical base of this work, and our goal is to identify different story elements within stories that conform with such a structure.

**Table 6:** A condensed version of the label definitions.

Class Label	Definition	Required Amount
Inciting incident	An event which motivates the main character and establishes the story.	0–3
Climax 1	A critical event that reveals what the story is **really** about.	at most 1
Obstacles	Any difficulties the main character encounters while he/she is pursuing his/her need.	0–5
Midpoint	An important event which pushes the story into a real **change**.	at most 1
Disaster	A terrible discovery—could be a consequence of the Midpoint.	at most 1
Crisis	A very bad or dangerous situation. The main character MUST make the ultimate choice.	at most 1
Climax 2	The event where the tension of the story conflict becomes the strongest.	at most 1
Climax 3	Shows the solution of the main conflict.	at most 1
Wrap-up	Reveals the situation after the solution was obtained.	at most 1
Non-Key	A less important scene	unlimited

The annotation task covered 61 episodes, in which there is one sample episode (season 1 episode 1) and 60 formal episodes. The sample episode was only used for practicing and concept familiarizing, and only the annotations of the formal episodes were included in the dataset. The 60 formal episodes are randomly divided into 20 groups (3 episodes per group), in addition to the sample episode. Each annotator was randomly assigned with one group and we ensured that there were at least three annotators for each group. Finally, only 3 episodes out of 60 (5%) had only 3 annotators, while all other episodes have more than 3 annotators.

### Appendix 3

#### Data over-sample

**Fig 6:**
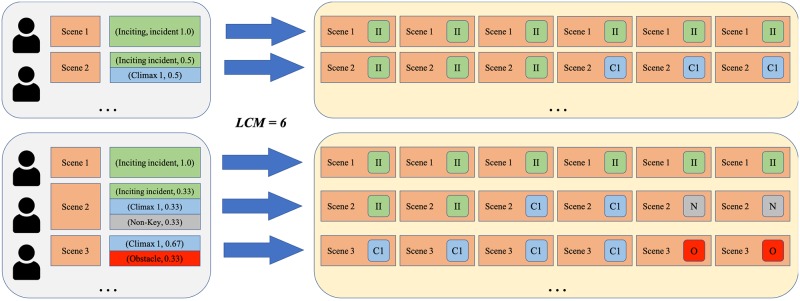
Illustrative example of the over-sample procedure. Assuming two episodes are annotated by 2 and 3 annotators, respectively: the least common multiple (LCM) of annotators for these two episodes is 6, thus we duplicate all scenes of these two episodes by 6, with *p*_*i*_ * 6 of them labeled with *l*_*i*_, where *l*_*i*_ is the *i*^*th*^ label in the annotation of a scene and *p*_*i*_ is the probability of *l*_*i*_.

As introduced in Section 3, the FSD dataset is composed of video scenes annotated by all possible labels with their corresponding probabilities; thus, there is a need to over-sample the data to make it suitable for multi-class classification, which requires one single label per record. Meanwhile, for each scene, the ratio of the probabilities of all possible labels is maintained by using the least common multiple (LCM) of the number of accepted annotators for each episode as the over-sample scale, see Fig 6 for example. With the LCM of 6, the two episodes are duplicated 6 times. Take scene 3 in the second episode for example, the annotation indicates that there is a probability of 67% (2/3) that this scene is a Climax 1, and 33% (1/3) that it is an Obstacle; therefore, after over-sampling, four duplicates of scene 3 are labeled as “C1” (Climax 1) and two are labeled as “O” (Obstacle). This over-sample strategy reflects the confidence extent of the annotators. For a given scene and its labels, higher confidence of the annotator leads to more data records after over-sampling, and, as a result, contributes more examples to be classified during the training stage.

## Supporting information

S1 File Full annotation guidelines. The guideline document we provided to the annotators in order to support them during the annotation task.
(PDF)

S1 Table Detailed label-wise performance. (PDF)
