# Supplementary material for: Towards story-based classification of movie scenes
Source: PLoS One. 2020 Feb 11;15(2):e0228579. doi: 10.1371/journal.pone.0228579 (PMC7012415; doi:10.1371/journal.pone.0228579)
Supplement: S1 File — The guideline document we provided to the annotators in order to support them during the annotation task. (PDF) [file pone.0228579.s001.pdf]

Scene Annotation Guidelines

Introduction

Usually a movie tells a story, and a story has its structure. This research aims at understanding a movie's narrative structure by identifying its key scenes and their roles in the movie's story. To train a scene classification algorithm, we need an annotated dataset. To accomplish the annotation task, you are asked to do the following:

1. Get familiar with the background concepts
2. Read the Scene Label Definition section **carefully** to understand the role of each key scene in the story
3. Read the Example section for better understanding of the definitions
4. Start the annotation task following the steps presented in Your Tasks section. The annotation tasks should be done in the Google forms provided in corresponding folders.

Background

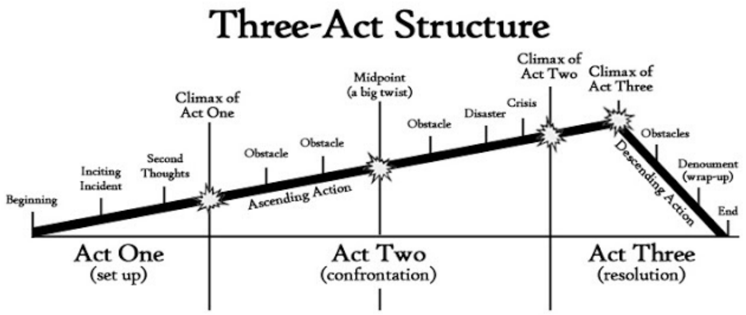

Figure 1: The typical structure of a movies' plot

In the movie industry, a movie is usually composed of **three "ACTs" (see the figure above)**:

- **ACT 1 is about Set-up**. The scriptwriter sets up the story, establishes characters, launches the dramatic premise (what the story is about), illustrates the situation, and presents the relationships between main characters.
- **ACT 2 is about Confrontation**. This ACT is all about conflicts between the main character's dramatic need (what he/she wants to win, gain, get, or achieve) and the obstacles he/she encounters.
- **ACT 3 is about Resolution**. This act presents the solution of the story: does the main character win or lose? Live or die? Get married or not? Return home safely or not?... The results of the main character's pursuing after his/her need are presented in this ACT.

Different key scenes in the movie play different roles to fulfill the tasks of the three ACTs. Your task is to identify the key scenes of a given movie and to annotate them with one of the class labels we provide. See the next section for detailed definitions of the key scene labels.

The movies you will need to annotate are from an American cartoon series: *the Flintstones*. It is a comedy series with four main characters: Fred & Barny and their wives Wilma & Betty.

Scene Label Definitions

Figure 1 in the previous section presents the key story elements, their possible location within a story and their sequential relationships. The following table presents the detailed definition of the key scenes. Though figure 1 gives hints to the sequential order of the labels (e.g., inciting incident, climax, obstacles, midpoint etc.), you do not have to follow this exact label sequence. For more detail see IMPORTANT NOTES below the definitions.

Class Labels	Definition	Example	Required Amount
**Inciting incident**	An event which sets the story in motion (motivates the main character) and establishes the story. It changes the main character's living situation and put him/her into a new world or new way of thinking.	Father murdered by the enemy in a revenge story / falling in love with a girl in a love story	0 - 3
**Climax of act 1**	A critical event that reveals the dramatic premise (what the story is <u>really</u> about) and the main character's commitment to a new way of behavior.	The hero decides to revenge the death of his father / The man decides to pursuit the girl	at most 1
**Obstacle**	Any difficulties the main character encounters while he/she pursuing his/her need.	Unable to arrive to an important location or get an important item etc.	0 - 5
**Midpoint**	Either a "false victory" or "false defeat". Something should happen here to push the story into a real change.	The hero seems to "win" but actually did not, or vice versa / The love between two seems to materialize but there are problems that they are not aware of	at most 1
**Disaster**	Opposite to the "Midpoint". If the midpoint was a false victory, this section will be a downward trajectory where things get consistently worse for the main character. If the midpoint was a false defeat, this section will be an upward trajectory where things get seemingly better.	The hero finds out the enemy is still there, and the situation is even worse / The problems revealing	at most 1
**Crisis**	A very bad or dangerous situation, but shows the main character's ultimate decision.	The hero almost fails / The lovers break up	at most 1
**Climax of act 2**	The main conflict, the main fight, or the main events for the main character.	The hero fight with his enemy / The lovers are trying to get together again	at most 1
**Climax of act 3**	A solution to the main conflict, shows the results of the main character's pursuing.	The hero finds the way to defeat his enemy / The lovers finally solved their problems	at most 1
**Wrap-up**	Reveal the situation after the solution was obtained.	The life of the hero after the fight / The life of the lovers after marriage	at most 1
**Non-Key**	If none of the above labels is applicable to the current scene, please use this label.		unlimited

IMPORTANT NOTES:

- The order matters! Although the order of the labels is not fixed, there is still a general structure of those labels.
    - In the annotation form, you will see climax 1, 2 and 3 as labels instead of climaxes of Act 1, 2 and 3. Because of the three-act structure, climax 1 should not appear in Act 2 and 3, the same for climax 2 and 3
    - Inciting incident could happen before climax 2
    - Obstacles could happen anywhere after climax 1
    - Midpoint should happen between climax 1 and 2, and also somewhere close to the middle of the story
    - Disaster should happen after the midpoint
    - Crisis could happen before or after climax 2
    - Wrap-up should happen after climax 3
- None of those labels is mandatory, meaning that some of the labels may not appear at all in an episode.
- Each scene can be only labeled with one of the above scene labels.

Example - Season 1 Episode 1: The Flintstone Flyer

In this section you will see examples of three scenes from the first episode of season 1 as well as their labels. **Before you read the examples, please watch the full episode.** You can find the video file of the sample episode (S1E1.mp4) in the folder named "sample" as well as the video clips representing their scenes in the sub-folder named "scenes".

The story of this episode: *Fred fakes illness so he and Barney can get out of taking their wives to the opera because they are going to take a bowl championship that night. However, the wives finally find out their little tricks.*

Sample annotations:

Scene ID	Scene Label	What happen in the scene	Reason of labeling
4	**Inciting incident**	In the previous scene, Fred was injured because of trying to drive the flying machine. In this scene, Fred tells Barney he will attend the bowling championship even though he is hurt.	The decision they made here motivates them to fool their wives later, and Fred's injure becomes their excuse. According to the definitions, this scene should be labeled as **inciting incident**.
6	**Climax of act 1**	In this scene, Wilma tells Fred the fact that they have planned for an opera night for months. But later on, Fred come up with the idea of faking illness.	The decision Fred has made in this scene is the key decision of the whole story. If he had not made this decision, then the rest of the story will not happen. According to the definition, this scene should be labeled as **climax 1**.

**10**	**Midpoint**	In this scene, Fred and Barney implements their plan and successfully won the trust from their wives.	It looks like they can have fun tonight in the bowling alley, but as we know from the story, this is just a "fake victory". According to the definition, this scene should be labeled as **midpoint**.

You need to annotate the rest scenes of the sample episode in order to get familiar with the concepts before starting the formal annotation. Detailed instructions will be given in the next section.

Your Tasks

1. Finish the annotation of the sample episode (in the previous section). During this step, ask immediately if you have any question regarding to the label definitions.
2. Annotate three episodes that are assigned to you. Annotation of each episode may take you approximately 1 hour. You can find the video files you need in the folder named "formal".

The annotation process for each episode should include the following steps (including sample and formal annotating):

1. Watch the episode **completely** and understand the story.
2. Answer a brief question for each episode. The questions can only be answered after you watch the episodes.
3. Repeat for each scene:
    a. Watch the video clip representing the scene in the folder named "scenes".
    b. Annotate the scene with a label from the list in Scene Label Definitions section.
4. After you finish one episode, do a self-check to see if your choices met all requirements in the next section.

Please remember:

1. Always think of the whole picture when you are not sure. You should keep in mind the whole story: what is it about, what is the main conflict, who is the main character in this episode and what does he want, etc.
2. You may find some consecutive scenes actually belong to one real "scene" in the original cartoons (presenting the same event). In this case, think about the real scene (i.e., all the consecutive scenes together) during the annotating process, if you think the real scene could be one of the labels, then choose the most representative scene in the experiment folder of that real scene instead of giving same label to all scenes. For example, if scene 10 to 12 belong to the same real scene in the cartoon, and you think the real scene is a Midpoint, then choosing the most important scene out of scene 10, 11 and 12 to be the Midpoint instead of the three of them.
3. You may find some scenes unable to open on google drive, or some scenes very short (e.g. less than one second). In these cases, label them as **Non-Key**.
4. While annotating the sample episodes, ask if there is ANYTHING you don't understand.

5. **Regarding editing your answers:** The annotation will be done within several google forms. Once you submit one form, you will receive a copy your current answers as well as a link for editing your submission. Make sure to keep the link safe otherwise you have to redo everything in case you want to change something.

Criteria of Acceptance

You need to fulfill all requirements below:

1. You **MUST** answer the questions in section 2 correctly (There is only one correct answer out of four). The questions are easy, so I will not tell you if your answers are correct or not. You can edit you answer at any time before the deadline. If you think the question itself is not clear, please ask me to explain.
2. The annotation of the three examples scenes (s1e1-4, s1e1-6, s1e1-10) should match their labels provided in the guideline.
3. The number of labels you selected in section 3 should meet the amount specified in the definition table. For example, according to the definition table, no more than 3 "inciting incidents" for one episode.
4. The first bullet in "IMPORTANT NOTES" below the definition table (about the order of labels) should be followed.

Once you think you have met all the requirements above, please send me an email **(with the title of: Final Evaluation + Name)** so that I can check the quality of your work.
