# Supplementary material for: Towards story-based classification of movie scenes
Source: PLoS One. 2020 Feb 11;15(2):e0228579. doi: 10.1371/journal.pone.0228579 (PMC7012415; doi:10.1371/journal.pone.0228579)
Supplement: S1 Table — (PDF) [file pone.0228579.s002.pdf]

S1 Table. Detailed label-wise performance. This table presents the label-wise performance for both metrics. The class labels and methods are abbreviated due to the length of the table. **Class labels:** I.I. - Inciting incident; C.1. - Climax 1; O. - Obstacle; M. - Midpoint; D. - Disaster; Cr. - Crisis; C.2. - Climax 2; C.3. - Climax 3; W. - Wrap-up; N.K. - Non-key. **Methods:** M.C.V - Most common vector; L.D. - Label distribution; L.R. - Logistic regression; T.L.D. - Temporal label distribution; XGB. - XGBoost with all features. Each value in this table means the metric score performed by a given approach on a given class. For the most common vector baseline, all values except for Non-key are undefined because the output label vectors are all 0s, leading to invalid values when computing the cosine similarity and KL divergence. For the same reason, we had to reject a few episodes for a few class labels, since there are some rare classes which might never be selected within an entire episode; the last row shows the number of episodes used for each class.

Cosine similarity										
	**I.I.**	**C.1.**	**O.**	**M.**	**D.**	**Cr.**	**C.2.**	**C.3.**	**W.**	**N.K.**
**M.C.V.**	undefined	undefined	undefined	undefined	undefined	undefined	undefined	undefined	undefined	0.84±0.02
**L.D.**	0.34±0.02	0.29±0.02	0.43±0.02	0.31±0.02	0.27±0.02	0.26±0.02	0.31±0.02	0.26±0.02	0.22±0.01	0.84±0.02
**L.R.**	0.40±0.02	0.31±0.02	0.44±0.02	0.32±0.02	0.29±0.02	0.28±0.02	0.34±0.02	0.29±0.02	0.25±0.01	0.85±0.02
**T.L.D**	0.59±0.04	0.46±0.04	0.49±0.04	**0.40±0.03**	**0.39±0.05**	**0.39±0.05**	**0.50±0.04**	**0.63±0.05**	**0.86±0.05**	0.84±0.02
**XGB.**	**0.67±0.06**	**0.59±0.06**	**0.52±0.04**	**0.42±0.06**	**0.42±0.07**	**0.38±0.06**	**0.52±0.06**	**0.65±0.07**	**0.89±0.06**	**0.90±0.02**
**KL divergence**										
**M.C.V.**	undefined	undefined	undefined	undefined	undefined	undefined	undefined	undefined	undefined	0.30±0.04
**L.D.**	2.09±0.12	2.53±0.13	1.66±0.11	2.37±0.12	2.65±0.14	2.72±0.13	2.34±0.12	2.75±0.12	3.06±0.12	0.30±0.04
**L.R.**	1.94±0.12	2.45±0.13	1.64±0.11	2.34±0.12	2.59±0.14	2.66±0.13	2.25±0.12	2.63±0.12	2.94±0.10	0.29±0.04
**T.L.D.**	1.37±0.17	1.73±0.14	1.46±0.14	**1.98±0.18**	**2.11±0.18**	**2.18±0.26**	**1.61±0.16**	**1.15±0.15**	**0.54±0.12**	0.30±0.04
**XGB.**	**1.37±0.17**	**1.73±0.14**	**1.46±0.14**	**1.98±0.18**	**2.11±0.18**	**2.18±0.26**	**1.61±0.16**	**1.15±0.15**	**0.54±0.12**	**0.30±0.04**
**Number of episodes**	60	60	60	60	59	58	60	60	59	60
